# Open versus endoscopic bone resection of the dorsolateral calcaneal edge: a cadaveric analysis comparing three dimensional CT scans

**DOI:** 10.1186/s13047-014-0056-3

**Published:** 2014-12-19

**Authors:** Klaus Edgar Roth, Ramona Mueller, Eike Schwand, Gerrit Stefen Maier, Irene Schmidtmann, Murat Sariyar, Uwe Maus

**Affiliations:** Center of Orthopedic and Trauma Surgery, University Medical Center of the Johannes Gutenberg University Mainz, Langenbeckstrasse 1, Mainz, 55131 Germany; Department for Neurosurgery, University Medical Center of the Johannes Gutenberg University Mainz, Langenbeckstrasse 1, Mainz, 55131 Germany; Institute for Medical Biometry, Epidemiology and Computer Science, Johannes Gutenberg University Mainz, Langenbeckstrasse 1, Mainz, 55131 Germany

## Abstract

**Background:**

It has been claimed that endoscopic calcaneoplasty offers some advantages over open techniques in the surgical treatment of Haglund’s deformity due to reduced postoperative complications like stiffness and pain. Bony over-resection places patients at risk of these complications. The resulting question with regard to the quantitative differences of the extent of the bone removed using these two techniques has not yet been answered. The purpose of the study was to determine the resection volume of calcaneal bone for open and endoscopic surgical techniques.

**Methods:**

16 feet obtained from body donors were operated on in equal parts using either open surgical or endoscopic techniques, with the technique selected on a random basis. High-resolution CT scans were obtained before and after the interventional procedure and analysed to obtain 3-D polygon models. Post-operative models were subtracted from pre-operative models to provide the volume change resulting from the intervention. This was then correlated with the bone mineral density (BMD) of the preparation.

**Results:**

The extent of bony resection was greater in open surgical techniques than in endoscopic approaches. The average volume of bone resection was 0.80 (±0.34) cm^3^ in the endoscopic group and 3.04 (±2.91) cm^3^ in the group that underwent open surgery. After adjustment for bone mineral density the extent of the resection was significantly larger (p = 0.018) in the group undergoing open surgery. The two groups did not differ significantly with regard to BMD (p > 0.1). The extent of the resection fell by 0.011 cm^3^ per 1 mg/cm^3^ areal bone mineral density, i.e., a slightly lower degree of bone resection was associated with a higher bone mineral density.

**Conclusions:**

Assuming that the resection volume was adequate to treat the patient’s complaints a smaller resection volume seen in our study using an endoscopic technique might lead to fewer postoperative complaints and faster recovery.

## Background

Prominence of the posterosuperior lateral process of the calcaneus is referred to as the Haglund’s deformity [1]. This deformity is sometimes combined with insertional tendinopathy of the Achilles tendon [2,3]. Chronic overuse is thought to produce a bony hypertrophy and bursitis as a consequence of external pressure. This repetitive pressure promotes Achilles tendinopathy, sub Achilles bursitis and pain [4].

Surgical treatment in Haglund’s deformity involves removal of the dorsolateral part of the calcaneus up to the insertion of the Achilles tendon and the inflamed retrocalcaneal bursa [5]. The degree of bone decompression obtained is seen as one of the most important outcome parameters since cases of treatment failure resulting from this procedure are related to inadequate osteotomy [6,7]. At the same time, some authors have claimed that an aggressive bone resection heightens the risk of weakening the Achilles tendon insertion, calcaneus fracture, stiffness, and ankle pain [5,8-10]. Recommendations with regard to the amount of bone resection vary widely [11-13].

It has been claimed that endoscopic calcaneoplasty, which has become a popular alternative to open techniques in surgical treatment of Haglund’s deformity, offers some advantages over open techniques due to reduced postoperative complications like stiffness and pain [7,14,15]. So far, it has not yet been sufficiently determined as to why this treatment offers advantages over conventional techniques. Based on the data found in the medical literature, it seems plausible that aside from the reduced trauma caused to the soft tissue with endoscopic resection, the volume of the resection of the calcaneus could also constitute an important factor.

The study presented here concerns itself with the open question of whether the extent of bone resection differs between the two techniques and thus might influence the outcome. Our hypothesis was that the application of the endoscopic technique would result in reduced extents of bone resection compared to the open surgical technique.

## Methods

**Figure 1:**
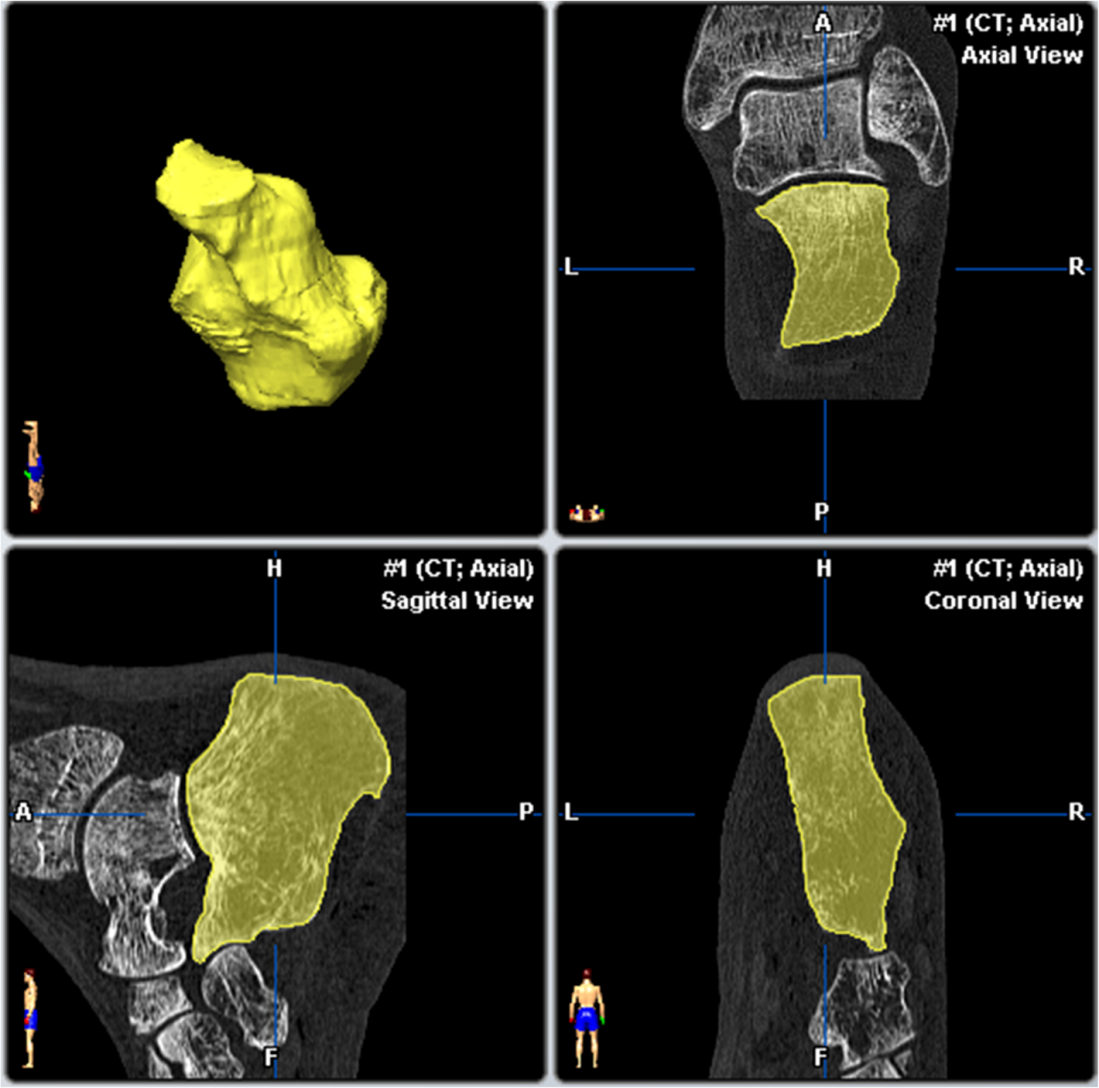
**Preoperative segmentation of the calcaneus facets.**

**Figure 2:**
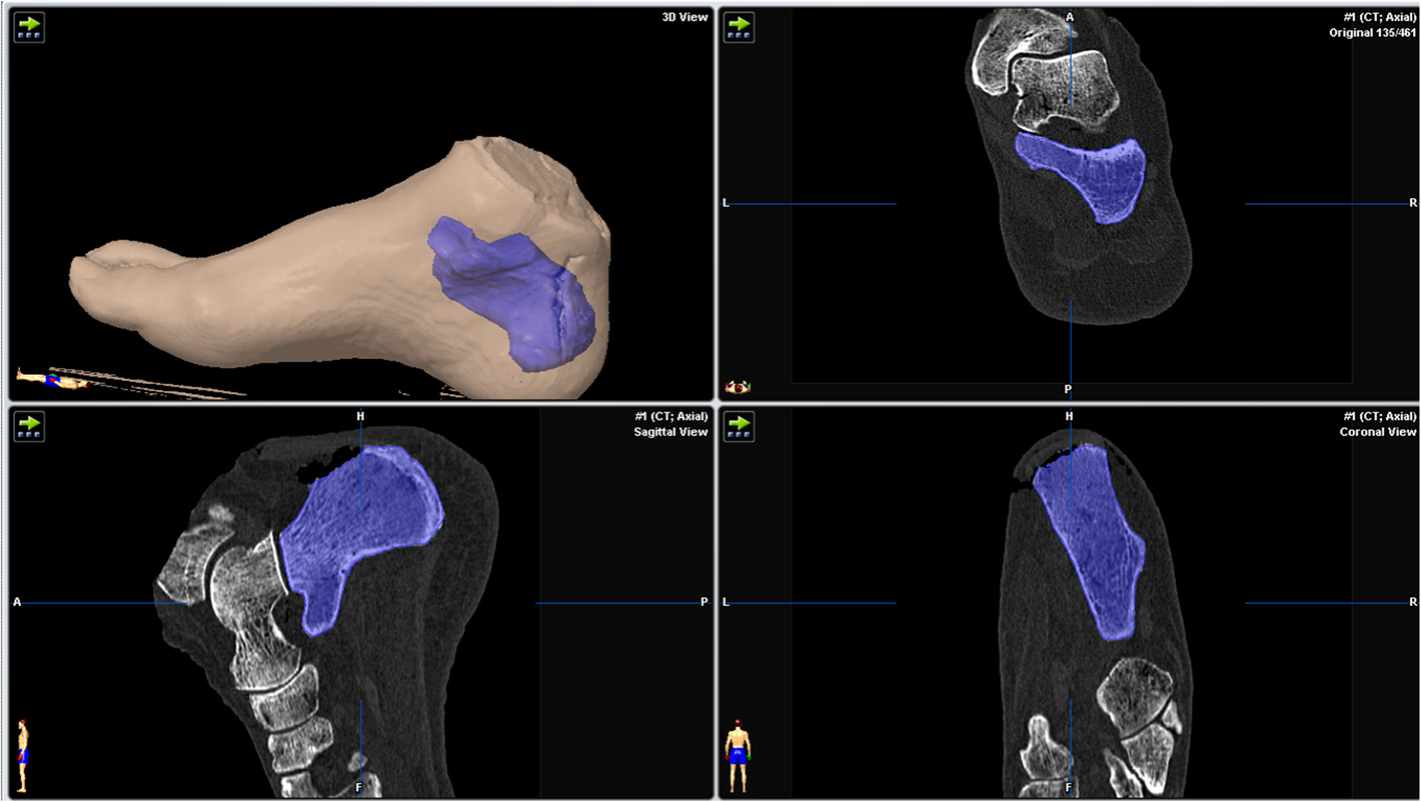
**Segmented representation of the calcaneus after calcaneoplasty.**

**Figure 3:**
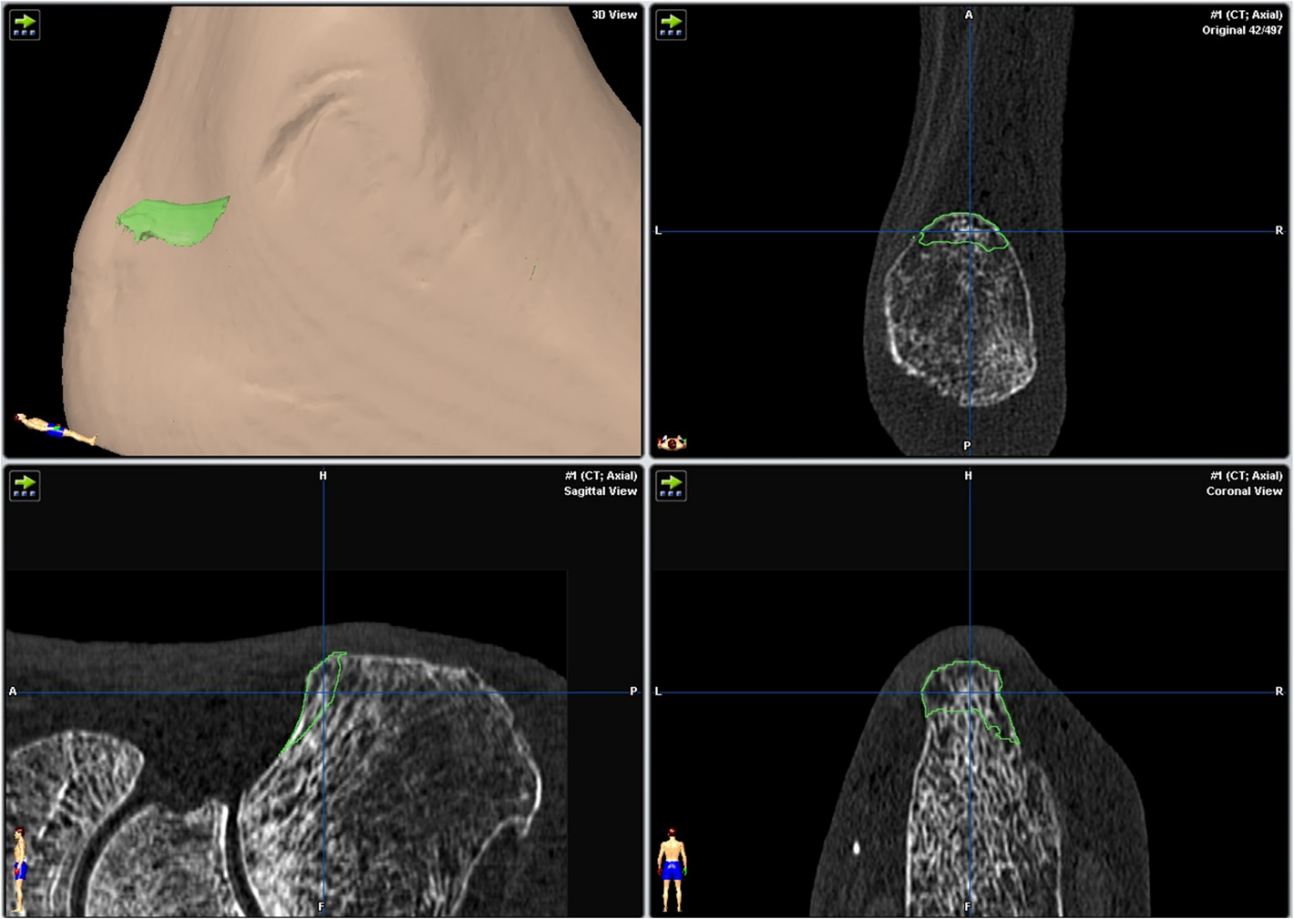
**Representation of the resected volume objects, spatial resolution of the course of resection.**

The study involved eight freshly frozen body donors. The use of cadaveric specimens was approved by the ethics committee of the national medical association Rhineland-Palatinate, Mainz, Germany. The specimens had no evidence of prior surgery or musculoskeletal disease. In each case both feet were separated above the malleoli and all sixteen preparations thawed at room temperature. These were then placed in the open or endoscopic groups on a random basis. A computerized random-number generator was used to formulate an allocation schedule. Subjects were randomized to either treatment with use of the method of randomly permuted blocks. The randomization scheme was generated with use of the web site www.randomization.com. Eight preparations were randomized into 2 blocks. Bone mineral density (BMD) was determined in the distal tibia using peripheral quantitative computed tomography (pQCT-XCT 2000, Stratec, Germany) and correlated with the dimensions of the resection. High-resolution CT data sets (Volume Zoom, Siemens Medical systems, Germany, Scanning protocol; Table 1) were obtained pre- and post-operatively. These were then converted to a 3-dimensional graphic representation using the Dextroscope planning system (Volume Interactions, Singapore) and the software iPlan (Brainlab AG, Feldkirchen, Germany). The calcaneus from every preparation was then segmented using “smart-brush” online tools under the same parameters and its volume was calculated. Subtraction of the post-operative from the pre-operative volume provided the volume of the resection with an accuracy of ± 0.01 mm^3^. A spatial representation of the resection was obtained by overlaying the pre- and post-operative data sets using the “image-fusion” function. The resected part of the calcaneus was displayed as a separate object volume (Figures 1, 2, and 3).

**Table 1:** **CT Scanning protocol**

**Resolution**	**UHR**
Centre/Window	50/1500
Filter	U70
Ink	0.8 mm
Collimation	4x0.75 mm
KV	120
mAs	100
Matrix	512
SD	1 mm

### Operative technique

Each preparation was fixed with a clamping device. All the procedures were performed by the author. The author is clinically experienced with both operations. Intra-operative x-ray control was used to visualize the extent of resection in both techniques.

Endoscopy: We implemented the 2-portal arthroscopic approach, with reciprocal access for the endoscope and the soft-parts or bone shaver (Arthrex, Naples, Fl. USA). We used a 4 mm arthroscope positioned in the retrocalcaneal space. After local debridement, the Achilles tendon was localized. Subsequently the calcaneal bone was exposed and resected with the 4-mm burr up to the distal insertion of the Achilles tendon. The resection started at one edge (medial or lateral) and was completed after changing the portals.

Open surgery: the access path (medial or lateral) was chosen randomly on the toss of a coin. The post-superior part of the calcaneus was exposed by a paratendinous incision. Hohmann retractors were positioned around the bony prominence and this was resected at an angle of approximately 45° with a 1 cm wide Simal-chisel. Bony remnants were then abraded with a rasp until no further protrusion was discernable by palpation.

### Statistics

The endpoint of the study was the resection volume resulting from the different operative procedures. Data are presented as means ± standard deviation. We used the Shapiro-Wilk-test to assess the normality assumption, which could not be rejected with our data. To address the main question regarding the resection volume, we used a linear regression model with resection as dependent variable and two independent variables: the continuous covariate ‘bone density’ and the binary variable ‘operative procedure’.

We performed a post-hoc power calculation in order to assess our ability to detect a difference between the operative procedures with the 16 preparations. When using a Satterthwaite *t*-test, we were able to find a resection volume difference of 2.2 between the operation procedures with a power of 80% and type I error of 0.05. *P*-values <0.05 were considered significant. All tests were two-sided.

## Results

**Figure 4:**
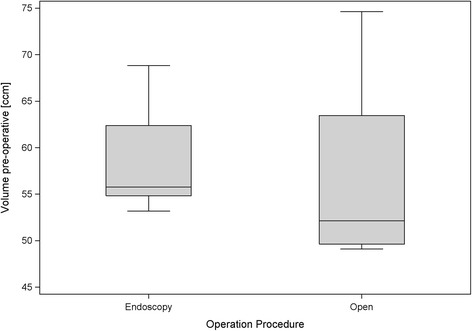
**Box plots for the preoperative calcaneus volumes for both procedures.**

**Figure 5:**
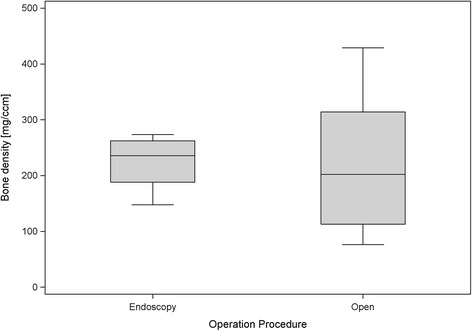
**Box plots of bone mineral density for both procedures.**

The average bone mineral density of the preparations was 222.25 (±96.42) mg/cm^3^, the mean pre-operative calcanear volume amounted 57.64 (±8.58) cm^3^ (Figure 4). The preparations that underwent endoscopic surgery had a bone mineral density of 224.02 (±47.94) mg/cm^3^ and an initial volume of 58.50 (±6.21) cm^3^, while the preparations that underwent open surgery had a bone mineral density of 220.34 (±132.72) mg/cm^3^ and an initial volume of 56.78 (±10.83) cm^3^. Thus it may be seen that randomization led to some differences between the bone mineral density and pre-operative volume of the two small groups. Nevertheless the levels remained analogous, as could be seen from *p* values greater than 0.1 that were obtained in the descriptive Mann–Whitney tests. The outlying values obtained from preparations 15 and 16 (Table 2) may be regarded as responsible for the higher standard deviation obtained from the preparations that underwent open surgery. This is also apparent from the corresponding box plot diagram (Figure 5).

**Table 2:** **Summary of the preparation’s characteristics and results**

**Preparation**	**Gender**	**Age**	**Operative technique**	**Calcaneus volume pre-operative (cm** ^**3**^ **)**	**BMD Calcaneus (mg/cm** ^**3**^ **)**	**Resection volume (cm** ^**3**^ **)**
1	M	63	1	55.57	268.40	0.56
2	F	79	1	55.90	273.40	0.42
3	F	87	1	54.11	249.90	1.27
4	F	69	1	53.18	256.20	0.77
5	F	75	1	56.84	160.10	1.10
6	M	81	1	55.64	147.70	0.64
7	M	84	1	68.84	221.00	0.49
8	F	59	1	67.93	216.70	1.19
9	F	93	2	50.05	223.40	0.54
10	F	86	2	52.68	225.70	1.13
11	M	77	2	73.66	428.90	1.67
12	F	72	2	74.64	402.20	1.91
13	F	79	2	53.26	180.50	2.61
14	M	85	2	51.59	137.90	2.47
15	M	86	2	49.22	76.10	4.33
16	F	89	2	49.10	88.00	9.67

1: endoscopic technique, 2: open technique; M = male, F = female.

**Figure 6:**
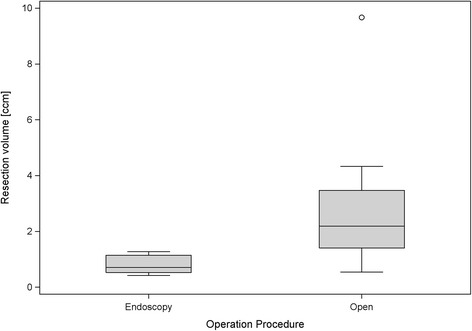
**Box plots for the volume of resected bone in both procedures.**

The average volume of bone resection was 0.80 (±0.34) cm^3^ in the endoscopic group and 3.04 (±2.91) cm^3^ in the group that underwent open surgery (Figure 6). When adjusted for bone mineral density it was clearly shown that the open surgery group had a higher amount of resection compared to the endoscopic group (*p*-value for the overall regression is 0.018; regression coefficient of the operative procedure is 2.19 (*p* = 0.031), and regression coefficient of the bone mineral density is −0.011 (*p* = 0.042)).

The extent of removal of the posterior-superior calcaneus in preparations 15 and 16 (8.8% and 19.7% of the preoperative bone volume respectively) was greater than that of the other preparations (on average 1.4% in the endoscopic group and 5.4% in the open surgery group).

The extent of the resection reduced by 0.011 cm^3^ per 1 mg/cm^3^ areal bone mineral density (i.e., a slightly lower degree of bone resection was associated with a higher bone mineral density).

## Discussion

The most important finding of our study is that the open surgical technique results in the resection of significantly more bone than the endoscopic approach. Ever since the endoscopic resection technique was introduced as an alternative treatment of Haglund’s deformity to the open surgical technique, two questions remained unanswered: Firstly, which of the two surgical procedures produces better treatment results in terms of patient satisfaction and secondly which parameters influence the outcome.

Adequate relief of symptoms is considered to be dependant on removing any osseous irritation to the Achilles insertion [16], as the degree of bone decompression obtained is seen as one of the most important factors of success [7,10]. In any event controversy remains over the exact amount of bone resection required in calcaneoplasty.

Recommendations as to the dimensions of resection are not evidence-based and vary widely. They range from an “oblique partial osteotomy starting approximately 1 cm anterior to the superior angle and angling downward to the insertion of the Achilles tendon” [12], to a resection of 1×2×2 cm [11] or 3×3×0.6 cm [8]. Sella et al. in his retrospective study of 16 heels highlighted the importance of enough bone being resected to allow decompression of the tendon and the retrocalcaneal bursa [17]. Schepsis and Leitze confirmed that most failures of this procedure are related to inadequate osteotomy [6,18]. In his retrospective study treating 35 heels, Nesse also recommended an “aggressive resection” [9].

A recently published review showed that the endoscopic approach produces superior results to an open approach (91% good or excellent results vs. 73%) on the basis of patient satisfaction and when comparing procedure-specific complications [7]. In this review, an increased complication rate was found consistently in the studies that evaluated open surgery (0.7% vs. 4.3% major complications). Regarding surgery outcomes, most authors included in the review stated that it was important for enough bone to be resected to minimize the risk of impingement and recurrence of complaints [7]. Bone over-resection, however, places patients at great risk of lesions of the Achilles tendon insertion, calcaneus fracture, stiffness, and ankle pain [5,8-10]. Despite these results, previous publications lack information regarding bone resections obtained with the different operative techniques.

The results of our study align with our initial study hypothesis and suggest that the advantages of the endoscopic surgical procedure lie in reducing bone trauma and resulting complications. Our interpretation of the results of this study is that the removal of that part of the post-lateral calcaneus that tends to impinge on the Achilles tendon can be better differentiated during endoscopy than with open surgery. Endoscopy facilitates more precise local decompression and thus avoids unnecessary resection of bony substance (i.e., preventable bone loss).

In our study the extent of bone resection was dependent on bone quality. While this fact is not surprising, it has not yet been taken into account in the context of resections in connection with Haglund’s deformity. The substantial bone loss witnessed in preparations 15 and 16 (8.8% and 19.7% of the initial volume of the calcaneus respectively), which underwent open surgery are hardly imaginable when applying an endoscopic treatment technique with the resulting more controlled resection. In this context, the greater precision offered by endoscopically assisted resection discussed earlier and the lower BMD of the two preparations are significant factors. In cases 15–16 the large amount of bone resected probably resulted from the chisel getting into a weak area or grain, leading to the removal of much more bone than intended. Given the better control over the extent of resection provided by a protected burr, its use would probably have avoided such outliers. But as this tool is not in widespread use in Europe in an open technique it was not used in the present study.

A weakness of this study is that it was performed on human cadavers not suffering from Haglund’s deformity. However different studies have shown that radiographic measurements of Haglund’s deformity have been neither very helpful nor reliable in predicting disease, or in planning treatment, due to numerous variations in the morphology of the calcaneus that have been described, especially for this posterior portion [2,9,17,19-21]. To this extent it would seem that despite the use of healthy body donors it remains legitimate to draw valid conclusions from our study. The repeated use of CT diagnostics employed in the present study is not applicable to patients on the grounds of radioactive exposure. Thus the present study did not involve patients with a verified Haglund deformity, but rather consecutive cadaver feet. Innovative radiological approaches such as Cone-beam CT (pedCAT, Curve beam Warrington, PA 18976, US) are in future, where applicable, capable of solving this problem. We consider it of negligible importance that the donated feet did not have a Haglund deformity, all the more so because this has no influence on the surgical techniques and the validity of the conclusions. The fact that both procedures were performed by the same surgeon can not be excluded as a further potential source of bias. In addition it cannot be ruled out that the reduced bone quality of the cadavers played a role in the results of the study.

## Conclusions

Our study revealed that using modern tools to resect the Haglund’s area of the calcaneus leads to much greater control leading to lower resection volumes. The results do not, however, reveal the cause of the differences in therapeutic outcomes between the operative approaches. But the technique described here allows, for the first time, precise quantification of the bone removed. Our aim in future studies is to correlate this parameter with clinical criteria in order to evaluate the actual relevance of bone resection on the clinical outcome. This will hopefully lead to faster recovery with fewer patient complications and greater patient satisfaction. This study has also shown that, regardless of technique, great care must be exercised when resecting poor quality bone and that bone chisels should not be routinely used.
